# Eating disorders and anabolic androgenic steroids in males - similarities and differences in self-image and psychiatric symptoms

**DOI:** 10.1186/1747-597X-8-30

**Published:** 2013-08-19

**Authors:** Tabita Björk, Kurt Skårberg, Ingemar Engström

**Affiliations:** 1 Department of Clinical Neuroscience, Psychiatry, Karolinska Institute, Stockholm, Sweden; 2 Psychiatric Research Centre, Örebro, Sweden; 3 School of Health and Medical Sciences, Örebro University, Örebro, Sweden

**Keywords:** Male eating disorders, Anabolic androgenic steroids, Self-image, Psychiatric symptoms

## Abstract

**Background:**

Body dissatisfaction is common among both females and males. Dissatisfaction with the body is a risk factor both for onset of eating disorders and for abuse of anabolic androgenic steroids (AAS). Few studies have however investigated if there are other similarities in respect to self-image or psychiatric symptoms between clinical samples of eating disordered males and males in treatment for negative effects of AAS use**.**

**Aim:**

The aim of this study was to compare two clinical samples, one of males with ED and one of males who used AAS, regarding self-image and psychiatric symptoms.

**Methods:**

This study compared males with eating disorders (n = 13) and males who recently stopped AAS use (n = 29) on self-image and psychiatric symptoms, using The Structural Analysis of Social Behavior self-questionnaire and a shortened version of The Symptom Check List.

**Results:**

The eating disorder group reported significantly lower scores for Self-emancipation and Active self-love and higher scores for Self-blame and Self-hate. Both groups reported serious psychiatric symptoms. The common denominator between groups was serious psychiatric symptomatology rather than negative self-image.

**Conclusions:**

The negative self-image profile, especially self-hate, found among males with Eating Disorders may indicate that the studied groups differ in aetiology of the underlying problems. The serious psychiatric symptoms in both groups call staff to pay attention to any thoughts of suicide due to severe depressive symptoms where by specialized psychiatric treatment may be needed.

## Background

Dissatisfaction with the body is very common in the population, in females in all ages [1] as well as among males [2]. Studies on female and male body image show the role of the media in defining and perpetuating body ideals [3], e.g., a muscular ideal male body type [4], or a thin female ideal [5]. A meta-analysis of the effects of the media on male body image concerns, yielded similar effect sizes as those found with women [6]. As a result of internalization of cultural norms, females become dissatisfied with the lower part of their bodies from the waist down and try to lose weight while males primarily want to change the shape of upper part of their bodies (stomach and chest) and are more likely to desire an increase in weight [7]. Body dissatisfaction has been reported as a risk factor, and one of the strongest predictors for onset of an eating disorder (ED) [8-10] and is also associated with low self-esteem and depression [8,11]. Dissatisfaction with the body seems to be the common and prominent denominator, not only between the sexes, but also between males with ED [12] and males using anabolic androgenic steroids (AAS) [13]. Both ED and the use of AAS may seriously affect physical health and the psychological and social wellbeing of those who suffer from those problems [14,15].

### ED among males

ED is a long-lasting mental disorder, characterized with disturbed eating or weight controlling behavior [14]. Studies have shown that ED are most common among young women, and that only about 10 per cent of patients with ED seen in mental health care are males [16]. Some have found that only 16 per cent of males with ED in fact seek treatment [17]. The proportion of males, 18 years or older in specialist treatment for ED in Sweden has been lower (1.5%) [18], than expected from prevalence studies [16]. A report from Swedish Quality registry on ED indicates an increase in the amount of males seeking treatment, since 4% of the adult patients in specialist ED-treatment in Sweden last year were males [19].

Earlier research studies on ED often excluded males and our knowledge about males with eating disorders is therefore still sparse [12,20]. However, research has begun focusing on males with ED and similarities between genders have been found, such as multi-factorial causes of ED, the core symptoms of ED, and suggestions that the course of illness, treatment response and long-term prognoses are comparable, which means that ED among males are also associated with an increased risk of mortality [21-24]. Differences between genders have however also been reported [25,26]. A history of premorbid overweight is more common among males with ED [23], and they report significantly lower scores on drive for thinness than females with ED, since they rather strive for a lean muscularity [27-29]. Males often use exercise as a compensatory method, while females vomit to control their body weight. Males are also more likely to binge eat than females [12,30-32]. Stanford and Lemberg [27] conclude that ED symptoms in males especially differ from females with ED in the construct of body dissatisfaction and the compensatory behavior associated with bulimia. Some results also indicate prognosis and outcome to be more favorable for males with shorter time to recovery and higher proportion of males reaching recovery [33].

### AAS among males

AAS, synthetic derivatives of the male endogenous sex hormone testosterone, were originally used by athletes but are now used by a far wider range of groups outside of sports and athletics [34,35]. The majority of AAS users are males [36-38]. In Sweden, between 50 000 – 100 000 people are thought to have used AAS, about 1% of the population of 9 million [39]. Lifetime prevalence of AAS use among males in USA is estimated to 0.9% and to 0.1% among females in the general population, while the prevalence of AAS use in Poland is 6% among males and 3% among females [40]. In Western countries life time prevalence of AAS in males ranges from 1% to 5%, and among females the prevalence is estimated to 0.1% [41]. The users reason for using AAS is to improve their appearance as well as performance [39,42]. Serious physical (i.e. cardiovascular, reproduction and endocrine system), psychiatric (i.e. depression, aggression and sleeping problems) [15] and social side effects (i.e. abuse of other drugs, battering of spouses and other criminality) [43] of AAS misuse have been reported. Heightened levels of violent behaviors are also reported among AAS-users [44].

Comparisons between males with ED, male bodybuilders and normal controls revealed that bodybuilders more closely resembled the ED group than normal controls regarding body dissatisfaction and loss of sexual desire [45]. Few studies have so far investigated why some of the body dissatisfied males become oriented towards thinness and why others become focused on muscularity. One study indicates that the groups may differ regarding body ideals [2].

### Are there other differences or similarities?

AAS use can be associated with body image disorders as “Muscle Dysmorphia” [46,47], sometimes also called “reverse anorexia nervosa”, which is defined as a fear of being too small [48]. The authors discuss the possibility that this “reverse anorexia nervosa” in males may be a similar disorder to anorexia nervosa in females and account for the lower prevalence rates of anorexia nervosa in males.

Other similarities found between males with ED and body-builders including AAS users were characteristics such as perfectionism, ineffectiveness and low self-esteem [42]. An essential question is whether more similarities can be found between males with ED and AAS users or if these groups differ in some essential respects. It is for example unclear whether there is a distinction between males with ED and males using AAS regarding the occurrence of underlying interpersonal profiles like negative self-image and the severity of psychiatric symptoms. Based on earlier studies showing several similarities between these groups, we anticipated that negative self-image and psychiatric symptoms would be similar between males with eating disorders and males who recently used AAS.

The aim of this study was to compare two clinical samples of males, one of males with ED and one of males who used AAS, regarding self-image and psychiatric symptoms.

## Methods

### Sample

Male adult ED patients (n = 13) and males who recently terminated use of AAS (n = 29) were included in the study. Data from the initial assessment at the start of treatment was used for both groups in the present study.

### Selection of the ED group and sample characteristics

The males with ED were collected from the Co-ordinated Evaluation and Research at Specialized Units for Eating Disorders (CO-RED) project. This naturalistic longitudinal project studied 840 adult patients seeking treatment at 14 specialized centers for ED across Sweden. All thirteen males who started treatment (1.5%) were included in the study. The males showed the typical ED psychopathology with weight phobia, binge eating, compensatory behavior and body image dissatisfaction, and were all diagnosed with an ED according to DSM-IV [49]: 3 (23%) had Anorexia Nervosa, 3 (23%) had Bulimia Nervosa and 7 (54%) were diagnosed with Eating Disorder Not Otherwise Specified. Age at onset of their ED varied between 8 to 19 years, and the average duration of the ED at treatment start was 11.5 years (Md). Age at treatment start was 20.5 years (Md) and varied between 18 to 35 years. None of the males in the ED group reported any history of drug abuse. Further information about the CO-RED project is described in more detail elsewhere [18].

### Selection of the AAS group and sample characteristics

AAS users were consecutively included from a doping clinic in an addiction centre (AC) in Örebro County. A total of 36 AAS users who recently terminated use of AAS were attending the AC to seek help for different AAS-related side effects. This included somatic, psychiatric, and/or social problems, for example gynecomastia, depression or relationship problems. At the AC they were all screened for psychiatric problems (including the presence of Eating Disorders or Muscle Dysmorphia). All AAS users were also screened for drug use several times. Twenty-nine of these patients (80.5%), all males were selected for the study. All 29 had used different AAS (human and/or veterinarian drugs), 28 (97%) used pharmaceuticals, 27 (93%) used narcotics, 13 (45%) used alcohol in a hazardous or harmful way and only one had never used narcotics or pharmaceuticals. They were all treated for different AAS related problems and diagnosed according to DSM IV [49]; one (3%) had Muscle Dysmorphia , two (7%) had ADHD , six (21%) had Anxiety Disorders, 10 (34%) were diagnosed with substance related disorders and 10 (34%) did not get any psychiatric diagnosis. In the somatic screening nine (31%) showed sexual related problems, five (17%) testicle atrophy, four gynecomastia (14%), five (17%) heart related problems (17%), 6 (21%) had acne, five (17%) liver related problems and six (21%) had striae problems. Eight (28%) of the 29 had no somatic problems. Twenty-eight (97%) were condemned for different crimes (e.g. crimes of violence, weapon offences or drug-related offences). The criminality in this sample has been studied and described in more detail elsewhere [50].

### Measures

*The Symptom Check List (SCL)* was used to measure self-reported psychiatric symptoms. A shortened, 63-item version of the SCL-90 [51] was utilised by removing the subscales for Phobic Anxiety, Paranoid Ideation, Psychoticism and Additional Scales.

*The Structural Analysis of Social Behavior (SASB),* (Intrex version, 3^rd^ surface, self-image) was used to assess self-image [52,53]. The questionnaire comprises 36 self-referential statements, some framed positively and others negatively. Responses are given on a scale from 0 to 100 with 10-point increments. Responses of 40 or above represent confirmation of the statement, whereas responses below 40 designate non-confirmation. The questionnaire forms eight clusters of self-image: (1) Self-emancipation, (2) Self-affirmation, (3) Active self-love, (4) Self-protection, (5) Self-control, (6) Self-blame, (7) Self-hate, and (8) Self-neglect. Cluster scores are obtained by dividing the sum of the items comprising the cluster by the number of items in the cluster. Recent empirical studies support the reliability of the SASB self-image questionnaire with a total Cronbach’s alpha = .74 [54].

The study protocol was approved by the Ethics Committee of Orebro County Council (No.: 538/99) and the Regional Ethics Vetting Board in Uppsala (No.: 2004: M-316) in accordance with the Swedish law concerning approval of medical research. The participants all gave their informed consent.

### Analysis

Data were analysed using SPSS for Windows version 17.0. Between-group comparisons were made using chi-square tests for categorical data, with Fisher’s exact test where appropriate. Independent two-tailed t-tests were used when comparing groups on age, Body Mass Index (BMI = kg/m^2^), dimensions of self-image and psychiatric symptoms, and effect sizes were computed for differences using Cohen’s *d,* in between-group comparisons [55]. We choose to show the exact p-value in all analyses.

## Results

Independent t-tests revealed that males with ED were significantly younger than the AAS group. An expected dissimilarity was also found regarding BMI; AAS group had significantly higher weight and BMI. The sample characteristics are shown in Table 1.

**Table 1:** Sample characteristics

**ED group (n = 13) M (SD) range**			**AAS group (n = 29) M (SD) range**		***p-value***
**Age**	22.7 (5.52)	18-35	26.1 (4.47)	19-36	0.04
**Height (m)**	1.78 (0.11)	1.60-1.98	1.79 (0.06)	1.64-1.90	0.73
**Weight (kg)**	63.5 (15.06)	38-95	98.8 (17.36)	62-140	0.01
**BMI**	21.0 (6.78)	14.36-41.33	30.8 (4.87)	21.13-43.06	0.01

Comparisons between groups on age, height, weight and BMI by independent t-tests.

### Between-group comparisons on self-image

**Figure 1:**
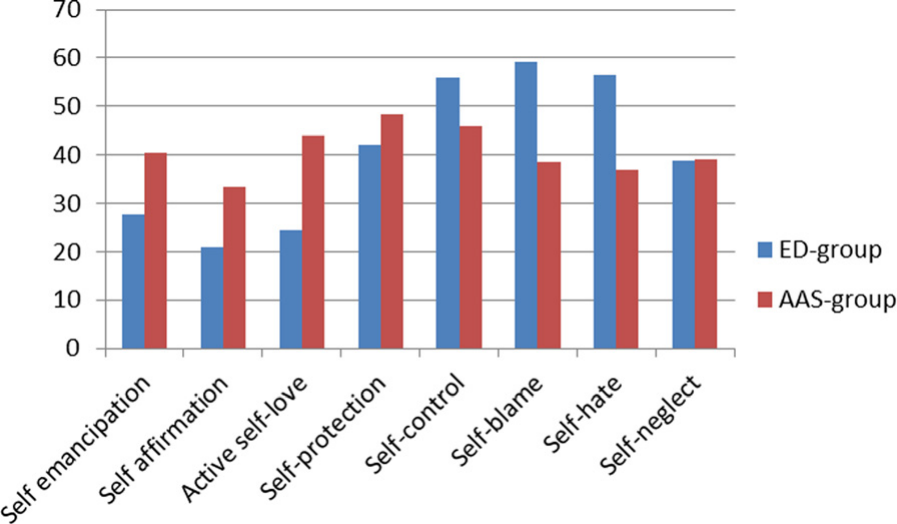
**Dimensions of self-image measured by SASB.** The distribution of mean values for each dimension of the SASB for the ED group and the AAS group. A normal self-image according to the SASB model is characterized by high values (≥ 40) on the positive clusters and low values (≤ 40) on the negative clusters.

Results from independent t-tests showed significant between-group differences with moderate to large effect sizes for differences between groups regarding self-image measured by SASB. The ED group reported significantly higher scores on Self-blame and Self-hate, as well as lower scores on Self-emancipation and Active self-love compared with the AAS group, which indicate a negative self-image in the ED group, but not in the AAS group (Table 2). Figure 1 illustrates the self-image profiles for the ED group and the AAS group.

**Table 2:** Self-image and psychiatric symptoms

**Measure**	**ED group mean (SD) at intake**	**AAS group mean (SD) at intake**	**Confidence Intervals of the difference**	***p*-value for *t*-tests**	***t-*value *(df)***	Effect size for comparisons d *
**SASB**						
Self emancipation	27.7 (11.5)	40.6 (17.2)	-23.5 - -2.25	.019	-2,45 (40)	.90
Self affirmation	21.0 (18.4)	33.4 (26.7)	-29.0 - 4.0	.135	-1,53 (40)	.55
Active self-love	24.5 (16.7)	43.9 (23.8)	-34.3 - -4.7	.011	-2,66 (40)	.96
Self-protection	42.1 (14.0)	48.2 (18.2)	-17.6 – 5.4	.292	-1,07 (40)	.38
Self-control	55.8 (21.4)	45.9 (19.4)	-3.6 – 23.4	.146	1.48 (40)	.50
Self-blame	59.2 (29.25)	38.5 (21.8)	4.3 – 37.1	.015	2,55 (40)	.81
Self-hate	56.5 (23.1)	36.9 (20.5)	5.3 – 34.0	.009	2,76 (40)	.90
Self-neglect	38.9 (19.6)	39.1 (23.6)	-15.3 – 15.1	.988	-,02 (40)	.01
**SCL-63**						
Somaticism	1.25 (0.7)	1.32 (0.82)	-.61 - .49	.813	-.24 (39)	.09
Obsession- compulsion	1.86 (0.8)	1.66 (0.93)	-.42 - .83	.514	,66 (39)	.23
Anxiety	1.59 (0.7)	1.65 (0.94)	-.67 - .55	.821	-,23 (39)	.07
Interpersonal sensitivity	1.69 (0.8)	1.46 (0.87)	-.36 - .82	.436	,79 (39)	.28
Depression	1.93 (0.8)	1.74 (0.88)	-.39 - .78	.504	,66 (39)	.23
Hostility	0.73 (0.7)	1.28 (1.12)	-1.26 - .16	.062	-1,93 (39)	.60

Between-group comparisons with two group independent *t*-tests from intake on the SASB and the SCL-63.

*d < 0.2 indicates no difference, *d =* 0.2-0.49 indicates a small difference, *d =* 0.5-0.8 indicates a moderate difference, and *d* > 0.8 indicates a large difference [55].

### Between-group comparisons on psychiatric symptoms

The results from independent t-test indicated no significant differences in psychiatric symptoms measured with SCL-63 between groups. Detailed results are presented in Table 2.

**Figure 2:**
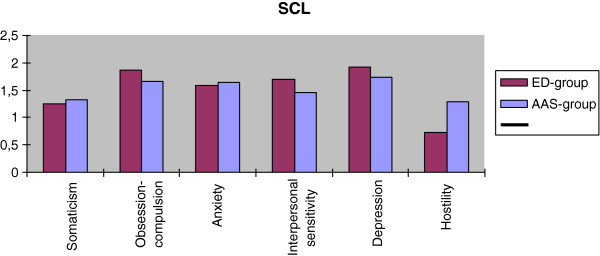
**Dimensions of psychiatric symptoms measured by SCL.** The distribution of mean values for the six dimensions of the SCL for the ED group and the AAS group.

Figure 2 illustrates the distribution of mean values and effect sizes for the differences between the ED group and the AAS group for each dimension of the SCL-63.

## Discussion

The present study aimed to compare self-image and psychiatric symptoms in males with ED and males who had used AAS. Our most important finding was that the ED group showed a considerably more negative self-image at treatment start than the AAS group, but the two groups were similar with respect to serious psychiatric symptoms. These results suggest that the common denominators between these two groups are serious psychiatric symptoms, particularly anxiety, depression, interpersonal sensitivity and obsessive-compulsive behavior rather than a negative self-image.

The negative self-image, especially self-hate, found among males with ED in this study is in line with other studies describing similar negative self-image profiles among females with ED [56]. Comparisons with published data for normal controls of self-image [56] indicate that our ED group differs considerably from normal controls on SASB on all dimensions of self-image, while the AAS group seems to differ most from normal controls with regard to less Self-affirmation. The typical feature of self-hate that was reported earlier among ED patients [56] is not that strongly marked in the AAS group, which may indicate that the aetiology of the underlying problems in these groups possibly will differ.

Different studies describe an association between psychiatric complications and use of AAS, e.g. mood changes and aggression [57] or even violence and, during AAS withdrawal, an association with depression [58]. AAS users in this study described that the psychiatric problems started or deteriorated after they started with AAS use.

This study points out the need to further examine these groups in a larger sample with a prospective design. Such design could determine whether the co-occurrence of psychiatric symptoms, earlier reported among body-builders using steroids [42] and in eating disorders [12], are an effect of body dissatisfaction with conflicts between internalization of cultural norms or if these symptoms are consequences of living with an ED or having a history of AAS use.

Clinical implications: These results highlight the need to detect these patients wherever they seek treatment. A challenge for health care is to reach these groups, and to intervene early before the consequences become severe. Results call staff to pay attention to any thoughts of suicide due to severe depressive symptoms. Their problems are of such severity that specialized psychiatric treatment may be needed.

### Limitations

Although this study shows significant differences in self-image between the ED- and the AAS group, and discloses severe psychiatric symptoms in both groups, it has certain limitations. The small sample size, reflecting the rarity of these clinical samples, limits the possibilities of generalization. However, males in specialist treatment for ED in Sweden are quite rare and the fact that this study included all males who were treated nationwide supports a representativeness of the ED group for treatment-seeking males with ED in Sweden. Another limitation concerns the selection of the AAS group. They were all seeking treatment for negative side-effects of steroid use and are therefore more likely to be representative of AAS users seen in health care, but not for those who may recently have started steroid use and predominantly experience positive effects [59]. A strength of this study is that it adds new pieces of knowledge not previously presented.

## Conclusions

This study highlights both the differences and similarities between the two groups studied: significantly different self-image profiles but similar psychiatric symptoms.

However, the serious psychiatric symptoms, equally prevalent in both groups, emphasize the need to examine the severity of psychiatric symptoms and offer psychiatric treatment for those in need, regardless of whether these psychiatric symptoms are a reason for the illness or a consequence of living with ED or AAS-use. It is important that more research focus on these specific groups, to ensure proper assessment and treatment. Future research needs to investigate further the role of self-image in prospective studies with larger groups showing ED or AAS use in males.

## Competing interests

The authors declare that they have no competing interests.

## Authors’ contributions

TB and KS conceived the idea for the study, participated in its design, took part in the analysis of results and drafted the manuscript. IE helped in the design of the study and was active in the analysis of results and helped to draft the manuscript. All authors read and approved the final manuscript.
